# Identification of PIEZO1 as a potential prognostic marker in gliomas

**DOI:** 10.1038/s41598-020-72886-8

**Published:** 2020-09-30

**Authors:** Wenjianlong Zhou, Xiangxiang Liu, Jan Willem Maurits van Wijnbergen, Linhao Yuan, Yuan Liu, Chuanbao Zhang, Wang Jia

**Affiliations:** 1 Department of Neurosurgery, Beijing Tiantan Hospital, Capital Medical University, Beijing, China; 2 Edwin L. Steele Laboratories, Department of Radiation Oncology, Massachusetts General Hospital, Harvard Medical School, Boston, MA USA; 3 Beijing Tongren Eye Center, Beijing Tongren Hospital, Capital Medical University, Beijing, China; 4 Bascom Palmer Eye Institute, University of Miami Miller School of Medicine, Miami, FL USA; 5 China National Clinical Research Center for Neurological Diseases (NCRC-ND), Beijing, China; 6 Beijing Neurosurgical Institute, Beijing, China

**Keywords:** Cancer, Computational biology and bioinformatics, Genetics

## Abstract

In multiple solid tumours, including gliomas, the mechanical properties change as the disease progresses. If and how mechanical cues regulate tumour cell proliferation is currently not fully studied. PIEZO1 has recently been identified as a crucial mechanosensitive cation channel in multiple solid tumours. However, we didn’t find any clinical data describing the association between PIEZO1 expression and glioma. To investigate the role of PIEZO1 in gliomas, we analysed PIEZO1 gene expression at the transcriptome level, genomic profiles and the association of PIEZO1 with clinical practice. In total, 1633 glioma samples with transcriptome data, including data from the Chinese Glioma Genome Atlas RNAseq, the Cancer Genome Atlas RNAseq and GSE16011 databases, were included in this study. Clinical information and genomic profiles including somatic mutations were also obtained. We found that PIEZO1 expression was highly correlated with malignant clinical and molecular subtypes of glioma. Gene ontology analysis showed that expression of PIEZO1 was correlated with tumour microenvironment-related genes that encode proteins involved in extracellular matrix (ECM) organization, angiogenesis and cell migration. Additionally, PIEZO1 was shown to be involved in tumour progression by serving as the central checkpoint of multiple ECM remodelling-related signalling pathways to modulate tumour cell proliferation and the tumour microenvironment in turn. Finally, high PIEZO1 expression was correlated with reduced survival time and acted as a robust biomarker for poor prognosis in gliomas. Taken together, the results indicated that high PIEZO1 expression is closely associated with highly malignant gliomas. Importantly, PIEZO1 serves as a key factor involved in sensing mechanical properties in the tumour and can regulate both tumour cells and their microenvironment to promote glioma progression, and it is also a potential therapeutic target for the treatment of gliomas.

## Introduction

The current gold standard treatment for glioma consists of chemotherapy, radiation therapy and surgery. Treatment strategies targeting the tumour microenvironment (TME) are gaining traction in the clinic^1^. The TME comprises all components surrounding a tumour; this includes blood vessels, immune cells, signalling molecules and extracellular matrix proteins. The TME interacts closely with tumour cells by providing nutrients and structural support, by communicating through the exchange of signalling molecules and often by promoting tumour growth and metastasis.

In fact, it has yet to be identified that apart from regulation, nutrition and structural support, mechanical stimuli sensed by tumour cells and neighbouring cells are also provided by the TME and affect every step of tumour progression and metastasis^2,3^. Tumour cells sense mechanical stimuli such as tissue pressure (stiffness), cell membrane tension, shear stress, and tissue pressure from the extracellular matrix (ECM). These mechanical cues are transduced into biochemical signals to modulate both tumour cells and the TME, promoting cancer progression^4^. These processes strongly depend on mechanosensitive ion channels.

PIEZO transmembrane proteins have been identified as mechanosensitive cation channels^5,6^. First described by Coste et al.^7^, homologues PIEZO1 and PIEZO 2 independently induce cationic non-selective activated currents in response to mechanical stimuli^8–11^. In breast cancer cells, PIEZO1 has been shown to increase motility in response to physical cues^12^, and PIEZO2 has been shown to play a role in the anchoring of brain metastatic cells^13^. As tumour cells grow, the mechanical forces within the tumour and TME increase, and the Piezo channel is activated by these mechanical signals and interacts with focal adhesions and integrin-FAK signalling to regulate tumour development^14^.

Glioma is the most prevalent primary malignant tumour of the central nervous system (CNS) in adults^15^. Despite advances in comprehensive care, the median survival time of patients who suffer from gliomas is only 15 months^16,17^. Prolonging the survival time is a critical challenge for clinicians treating glioma patients. In the past decade, there has been a surge in studies investigating molecularly targeted therapies and immunotherapies for the treatment of gliomas. However, interindividual tumour heterogeneity appears to temper the success of these studies^18^. It has become increasingly apparent that tumours do not result from the unregulated growth of a single cell but that tumour formation involves bidirectional abnormal communication between tumour cells and their microenvironment^19^. This not only plays an essential role during tumour progression and metastasis but also has a profound impact on therapeutic efficacy^20^. Additionally, recent studies addressed a feedforward circuit mediated by PIEZO1 and tumour tissue mechanics to promote glioma growth^21^. However, until now, the relationship between PIEZO1 expression and glioma malignancy in human patients has not been studied.

Transcriptomes from 325 gliomas from the Chinese Glioma Genome Atlas (CGGA) dataset were analysed to investigate the expression of PIEZO1 in gliomas, and we further verified these results by analysing RNA data from The Cancer Genome Atlas (TCGA) network derived from 1032 gliomas and a GSE16011 microarray of 276 cases. Moreover, genomic profiles containing somatic mutations were also analysed in our study. This is the first integrative study characterizing PIEZO1 expression in whole grade glioma both molecularly and clinically. Our work aims to improve the current knowledge of the role of mechanical forces in tumour aggressiveness mechanisms and provide a potential therapeutic target for gliomas.

## Materials and methods

### Data collection

The present study was approved by the Beijing Tiantan Hospital Institutional Review Board (IRB)/Ethics Committee of Beijing Tiantan Hospital. In total, 325 RNAseq samples were included in the CGGA dataset. We collected the molecular and clinical data from the CGGA database. The IDH mutation status detection method was described in Guan et al.^22^. mRNAseq data obtained from 1032 WHO grade II to grade IV gliomas were downloaded from The Cancer Genome Atlas (TCGA) (https://portal.gdc.cancer.gov/). We included 276 cases from the GSE16011 cohort. The RNAseq data used to study the specific tumour anatomic structure in GBM was collected from the Ivy Glioblastoma Atlas Project (http://glioblastoma.alleninstitute.org/).

### Bioinformatic analysis

We obtained 741 cases with somatic mutations and associated with RNAseq data from the TCGA database. We used GISTIC 2.0^23^ to evaluate the copy number alternations corresponding to PIEZO1 expression. The thresholded copy number at the alteration peaks was determined by GISTIC analysis (all_lesions.cof_99.txt file)^24^. The gene ontology (GO) analysis in DAVID Bioinformatics Resources 6.8 was used to explore the biological functions of the related genes^25^. Gene set enrichment analysis (GSEA) was performed as presented in Li et al.^26^. GO analysis of the most highly correlated genes was used to generate a heatmap after Spearman correlation analysis. The GO gene set was downloaded from MSigDB (https://software.broadinstitute.org/gsea/msigdb/index.jsp). ECM-related metagenes were described in the work of Rody and Jain^27,28^. Metagene expression values were obtained by quantifying the normalized mean expression values of all genes in their respective cluster^28^.

### Immunofluorescent staining and haematoxylin and eosin staining

Twenty Paraffin-embedded human glioma tissues for validation (10 LGG samples, WHO grade II–III diffuse astrocytoma; 10 GBM samples, WHO grade IV glioblastoma), normal human brain and mouse retina tissues were collected for staining. We reviewed the tissue slides through the visual evaluation of haematoxylin and eosin (HE)-stained slides. The results of the morphologic identification were independently confirmed by two neuropathologists. We selected a PIEZO1 antibody (NOVUS; NBP1-78537, 1:200) to detect PIEZO1 expression. Immunofluorescent (IF) staining of paraffin sections was performed. Briefly, specimens were blocked in blocking buffer for 60 min, the sections were incubated with primary antibody overnight at 4 °C, and then the sections incubated with the corresponding secondary antibody (Alexa Fluor 488, 1:200; DAPI, 1:1000) at room temperature in the dark for 1 h. After washing with PBS buffer, the slides were coverslipped with Prolong Gold Antifade Reagent. All images were analysed using Image-J based on the area fraction or intensity of positive staining.

### Statistical analysis

All statistical analyses were performed using the R programming language (version 3.4.1, https://www.r-project.org/) and ImageJ (version 1.52K, National Institutes of Health, USA). Spearman correlation analysis was used to evaluate the correlations between continuous variables. The differences in variables between groups were assessed by Student’s t-test, one-way ANOVA, or Pearson's Chi-squared test. We used a Kaplan–Meier survival curve to determine the survival distributions and performed a log-rank test to assess the statistical significance. Univariate and multivariate Cox proportional hazard models were determined to evaluate the prognostic value of PIEZO1. Patients with missing information were excluded from the statistical analysis. All statistical tests were two-sided. A *p* value < 0.05 was considered significant.

## Results

### Associations of PIEZO1 expression with clinical and molecular characteristics in gliomas

**Figure 1:**
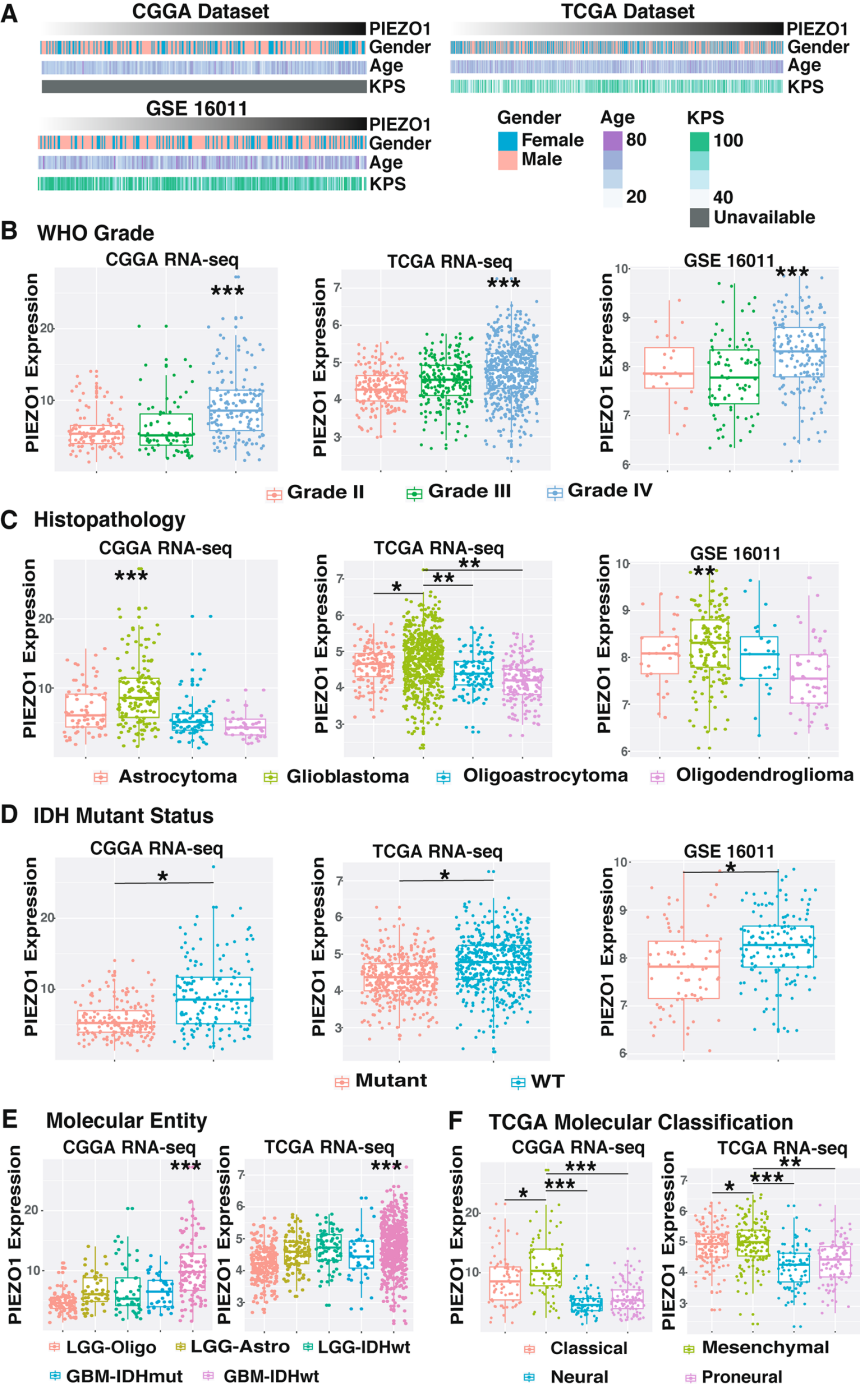
The landscape of clinical and molecular features in associations with PIEZO1 expression. (**A**) Datasets were arranged in order of increasing PIEZO1 expression. The relationships between PIZEO1 expression and clinical characteristics were evaluated; (**B**): PIEZO1 expression was upregulated along with the increasing of the WHO grade. *** On the Grade IV bar means the difference of expression of PIEZO1 between Grade IV and Grade II, Grade III respectively; (**C**) The expression levels of PIEZO1 increased by the histopathologic classification, *** and ** on the Glioblastoma bar means the difference of expression of PIEZO1 between Glioblastoma and Astrocytoma, Oligoastrocytoma, Oligodendroglioma respectively; (**D**) The expression levels of PIEZO1 increased in the IDH-wt gliomas compared with the IDH mutant gliomas; (**E**) The level of PIEZO1 expression in the molecular entity, *** on the GBM-IDHwt bar means the difference of expression of PIEZO1 between GBM-IDHwt and LGG-Oligo, LGG-Astro, LGG-IDHwt, GBM-IDHmut respectively; F: The PIEZO1 expression pattern in the TCGA molecular classification. ***p < 0.001, **0.001 < *p* < 0.01, *0.01 < *p* < 0.05.

Gliomas from the RNAseq and microarray sets were arranged in order of increasing PIEZO1 expression (Fig. 1A). PIEZO1 expression was evaluated among the groups and in association with factors including age and the KPS score, although we did not identify any difference. According to the histopathological heterogeneity of glioma, the RNA sequencing data was analysed based on histology, the WHO grading system and IDH mutation status. PIEZO1 expression was significantly higher in glioblastoma (WHO grade IV) than in low grade gliomas (WHO grade II and WHO grade III, Fig. 1B). With regard to histopathologic classification, glioblastoma had the highest expression of PIEZO1 (Fig. 1C).

Studies have incorporated the use of IDH mutations, 1p/19q codeletions and the TERT promoter as important alterations for glioma classification^24,29,30^. Here, we found that PIEZO1 was overexpressed in wild-type IDH-expressing gliomas, where it played an essential role in promoting cell viability and the aggressiveness of gliomas (Fig. 1D). Furthermore, the 1p/19q non-codeletion was associated with higher PIEZO1 expression (Fig. S1A). However, based on the TERT promoter status, there was no significant difference between the mutant and the wild-type in terms of PIEZO1 expression (Fig. S1B).

**Figure 2:**
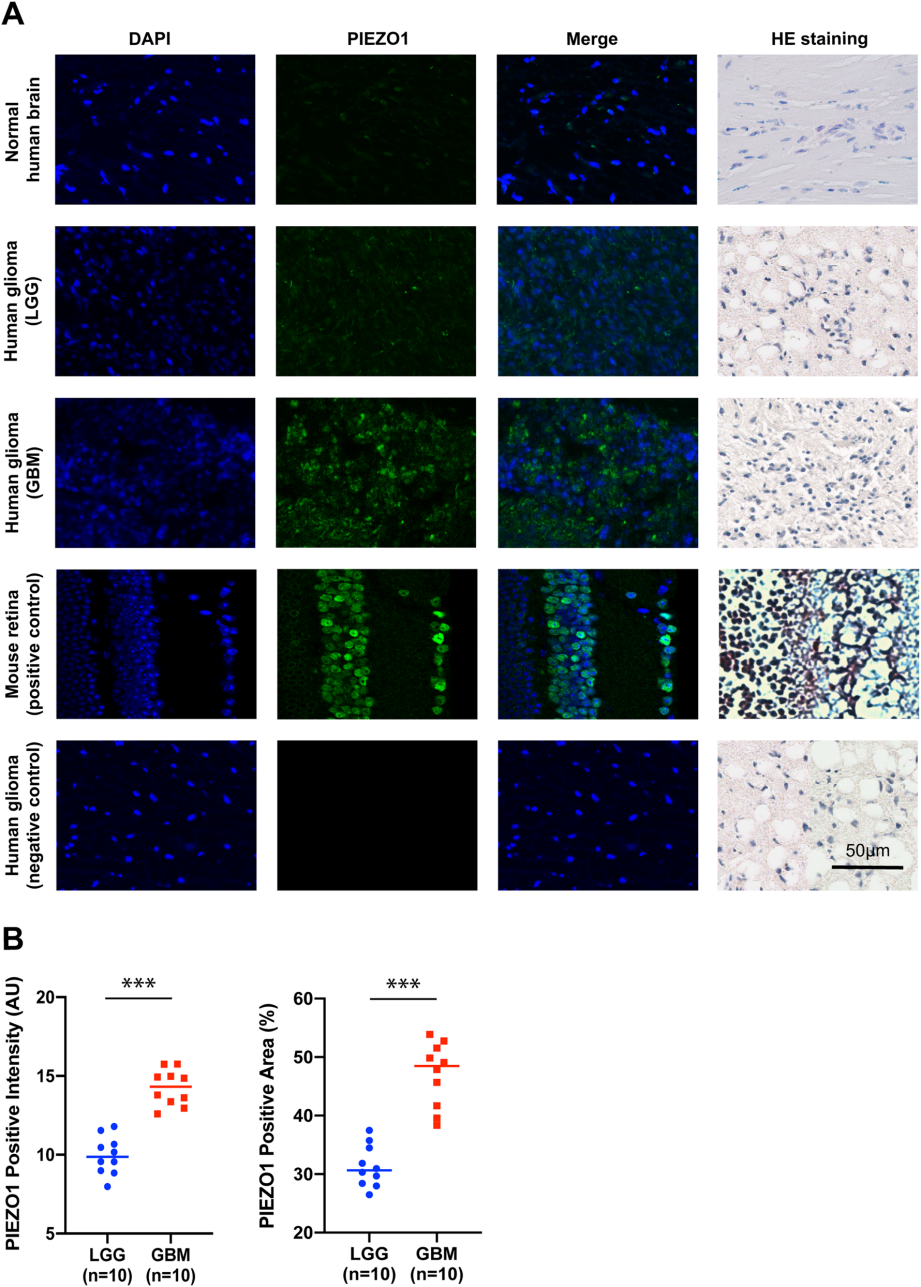
Immunofluorescence staining of PIEZO1. (**A**) IF staining of PIEZO1 in glioma tissues and normal human brain tissues; (**B**) Qualification of area fraction and intensity of positive PIEZO1 cells in LGG and GBM. PIEZO1 was highly expressed in GBM, compared to LGG. ***p < 0.001. Scale bar, 50 µm.

Molecular classification of gliomas has presented a new method for defining patient prognosis using genomic and transcriptomic data. Based on the 2016 WHO classification of tumours in the central nervous system, gliomas were divided into five molecular groups for further evaluation, which included lower-grade glioma (LGG)-oligodendroglioma (LGG-Oligo, IDH-mutant LGG with TERT promoter mutation or 1p/19q codeletion), LGG-astrocytoma (LGG-Astro, IDH-mutant LGGG without TERT promoter mutation or 1p/19q codeletion with ATRX mutation), LGG with wild-type IDH status (LGG IDH-wildtype), glioblastoma (GBM) with mutant IDH status (GBM-IDHmut), and GBM with wild-type IDH status (GBM-IDHwt). The expression of PIEZO1 was the highest in GBM-IDHwt and the lowest in LGG-Oligo (Fig. 1E). Gliomas can be categorized into four distinct molecular subtypes: classical, mesenchymal, neural, and proneural (PN). As shown in Fig. 1F, PIEZO1 was significantly upregulated in the mesenchymal and classical subtypes compared to other subtypes in both the TCGA and CGGA datasets. These results suggest that PIEZO1 may play a critical role in the progression of glioma. IF and HE analysis confirmed that the expression of PIEZO1 was upregulated in GBM (Fig. 2A,B). Choi et al.^31^ found astrocytes in the optic nerve head expressing multiple putative mechanosensitive channels, especially the recently identified channel PIEZO1. We used mouse retina as a positive control in our study.

In addition, primary and recurrent glioma in the same patients showed similar PIEZO1 expression (Fig. S1C). In GBM, there’s no significant difference in PIEZO1 expression between primary GBM and recurrent GBM. Meanwhile, the PIEZO1 expression was significant lower in secondary GBM compared to primary and recurrent GBM, respectively (Fig. S1D).

### PIEZO1 expression is associated with distinct patterns of genomic alterations

**Figure 3:**
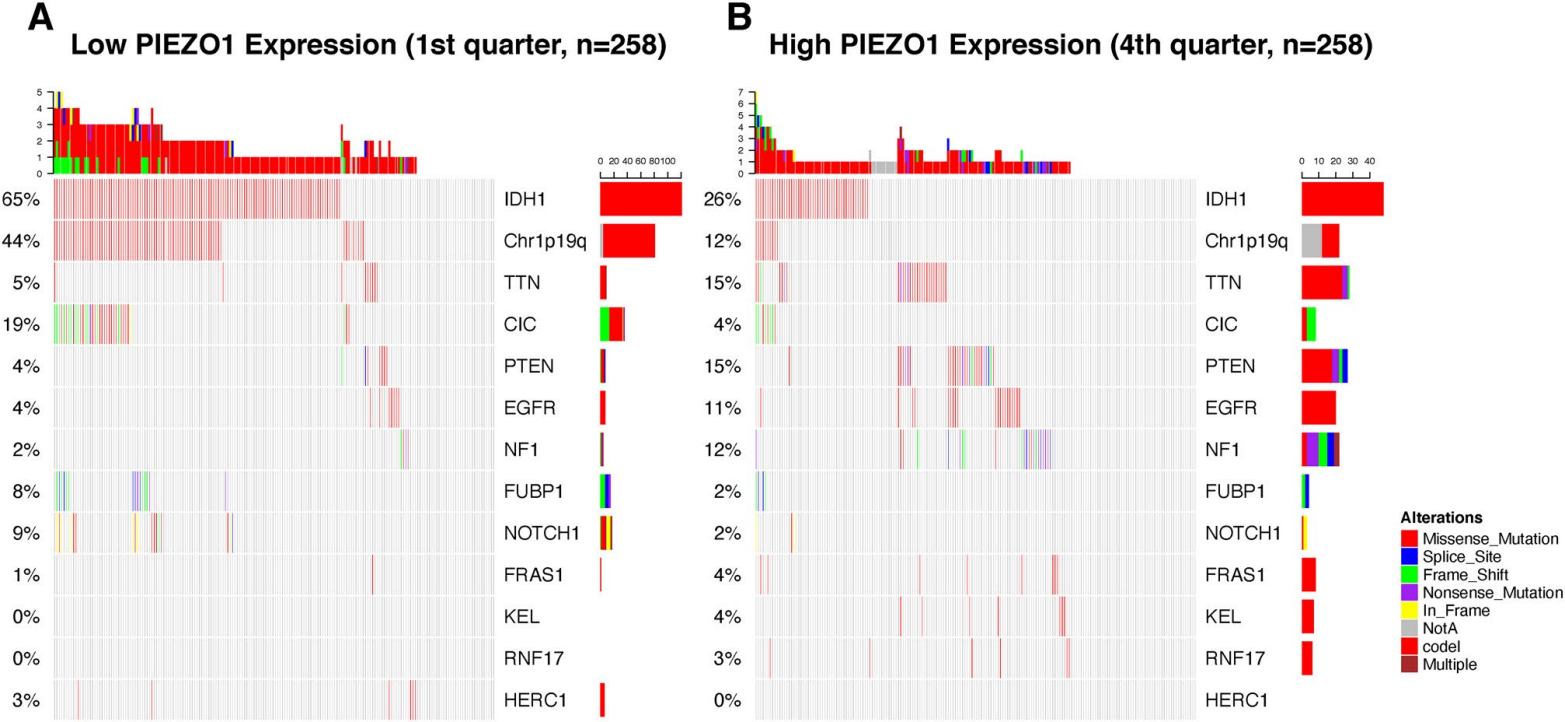
Distinct genomic profiles associated with PIEZO1 expression. (**A**) Differential somatic mutations were detected in gliomas with low PIEZO1 expression; (**B**) differential somatic mutations were detected in gliomas with high PIEZO1 expression.

The available somatic mutation information in the TCGA database was analysed to explore the molecular mechanisms in gliomas. Cases were classified into two, three, or four groups based on the order of increasing PIEZO1 expression. Parallel analyses were applied to each type of grouping to enhance the robustness of our findings. We dichotomized the patients into two groups based on the level PIEZO1 expression and compared the frequency of mutations between the high expression group and the low expression group. A high frequency of mutations in IDH1 and Chr1p19q was detected in gliomas with low PIEZO1 expression (Fig. 3A), while mutations in TTN, PTEN and NF1 were significantly enriched in cases with high expression of PIEZO1 (Fig. 3B). In addition, a significant difference in the frequency of mutations in CIC, EGFR, FUBP1 and NOTCH1 was detected in response to differences in PIEZO1 expression. The frequency of mutations in IDH1 and Chr1p19q was in accordance with our findings based on RNAseq.

### PIEZO1 indicates worse prognosis in gliomas

**Figure 4:**
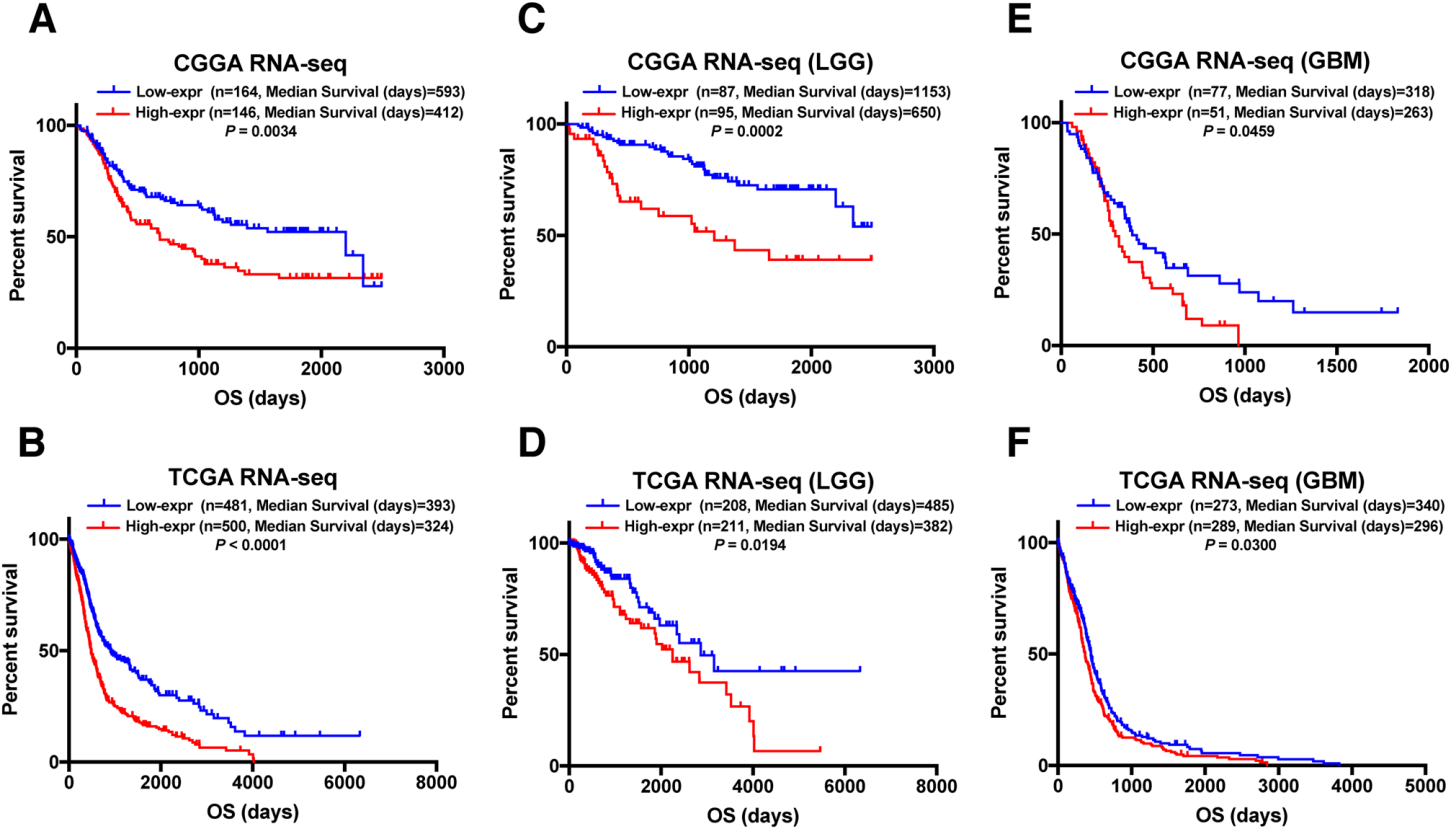
Clinical outcome in patients with gliomas in different PIEZO1 expression. (**A**,**B**) Different PIEZO1 expression conferred significantly different prognosis in all cases. (**C**–**F**) The prognostic value of PIEZO1 expression in different grades. Kaplan–Meier survival analysis was performed. Blue lines show the survival curve in patients of low PIEZO1 expression; Red lines show the survival curve in patients of high PIEZO1 expression.

Since PIEZO1 showed a positive correlation with poor outcome, we further used Kaplan–Meier analysis and the Cox proportional hazard model to investigate the prognostic value of high PIEZO1 gene expression. Patients with tumours expressing PIEZO1 at high levels generally had a significantly shorter OS than those with lower expression (Fig. 4A,B). Moreover, similar results were found in most patients in our dataset (Fig. 4C–F). Survival curves were also generated for the different subtypes and categorized according to the 2016 WHO molecular classification, IDH mutant status and histopathologic classification (Supplemental Fig. 2). Furthermore, we performed Cox regression analyses to explore the prognostic value of PIEZO1 expression in gliomas. Univariate analysis showed that the overall survival was significantly associated with PIEZO1 expression, malignant histopathology, high WHO grade, age at diagnosis, classical and mesenchymal subtypes and IDH mutations in CGGA dataset and TCGA dataset (Table 1). Moreover, univariate Cox regression indicated that PIEZO1 expression and the factors mentioned above were independent prognostic indicators for gliomas in the TCGA dataset, however, multivariate Cox regression failed to present similar results (Table 2). These results suggested that PIEZO1 may act as an indicator for poor prognosis in gliomas.

**Table 1:** Clinical and molecular characteristics of patients included in this study.

Cohort	CGGA-RNAseq	TCGA-RNAseq	GSE16011
	(n = 325)	(n = 1032)	(n = 276)
**Age, years**			
Mean (range)	43 (8–81)	50 (10–87)	51 (14–81)
**Sex**			
Female	122	400	88
Male	203	562	180
Unavailable	0	70	8
**KPS**			
Mean (range)	–	81 (20–100)	81 (0–100)
Unavailable	325	376	0
**Primary or recurrent**			
Primary glioma	240	0	0
Recurrent glioma	61	0	0
Secondary GBM	34	0	0
Unavailable	0	1032	276
**WHO grade**			
Grade II	109	216	24
Grade III	72	241	85
Grade IV	144	505	159
Unavailable	0	70	0
**Histology**			
Oligodendroglioma	40	159	52
Oligoastrocytoma	73	109	28
Astrocytoma	68	151	29
Glioblastoma	144	562	159
Normal brain	0	0	8
Unavailable	0	51	0
**Molecular classification**			
LGG-Oligo	45	234	0
LGG-Astro	87	107	0
LGG, IDH-wildtype	49	78	0
GBM, IDH-mutant	39	36	0
GBM, IDH-wildtype	105	526	0
Unavailable	0	51	276
**TCGA subtype**			
Neural	81	76	0
Proneural	102	83	0
Classical	74	738	0
Mesenchymal	68	135	0
Unavailable	0	0	276
**IDH status**			
Mutant	171	456	82
Wild-type	154	454	139
Unavailable	0	122	55
**1p/19q status**			
Codel	0	156	0
Non-codel	0	853	0
Unavailable	325	23	0
**Overall survival, days**			
Median	770	353	–
**Survival status**			
Alive	168	478	–
Dead	142	503	–
Unavailable	15	51	276

**Table 2:** Univariate and multivariate cox analyses in gliomas.

Factor	CGGA set					TCGA set				
	No. of samples	Univariate		Multivariate		No. of samples	Univariate		Multivariate	
		*p* value	HR	*p* value	HR		*p* value	HR	*p* value	HR
**Age**										
Increasing years	325	< 0.0001	1.038	0.446	1.006	1047	< 0.0001	1.059	< 0.0001	1.026
**WHO grade**										
Benign progress	325	< 0.0001	0.171	< 0.0001	0.22	1047	< 0.0001	0.138	< 0.0001	0.207
**Histopathology**										
Astrocytoma	66	< 0.0001	0.31	0.537	0.859	169	< 0.0001	0.192	0.743	0.927
Oligoastrocytoma	67	< 0.0001	0.163	0.007	0.466	114	< 0.0001	0.121	0.01	0.511
Oligodendroglioma	39	< 0.0001	0.022	< 0.0001	0.068	174	< 0.0001	0.11	0	0.068
Glioblastoma	138	Ref.	Ref.	Ref.	Ref.	590	Ref.	Ref.	Ref.	Ref.
**TCGA molecular classification**										
Classical	70	< 0.0001	2.839	0.802	1.08	105	0.106	0.788	0.015	0.688
Mesenchymal	65	< 0.0001	4.946	0.093	1.714	117	0.317	0.866	0.334	0.863
Neural	76	0.009	0.458	0.158	0.618	70	0.188	0.806	0.023	0.69
Proneural	99	Ref.	Ref.	Ref.	Ref.	73	Ref.	Ref.	Ref.	Ref.
**IDH status**										
Wild-type versus mutation	310	< 0.0001	4.101	0.298	1.337	926	< 0.0001	6.56	0.007	1.737
**PIEZO1 expression**										
Increasing expression	325	< 0.0001	1.128	0.988	1	1047	< 0.0001	1.627	0.076	1.027

### Overactivated signalling pathways were correlated with the expression of PIEZO1

**Figure 5:**
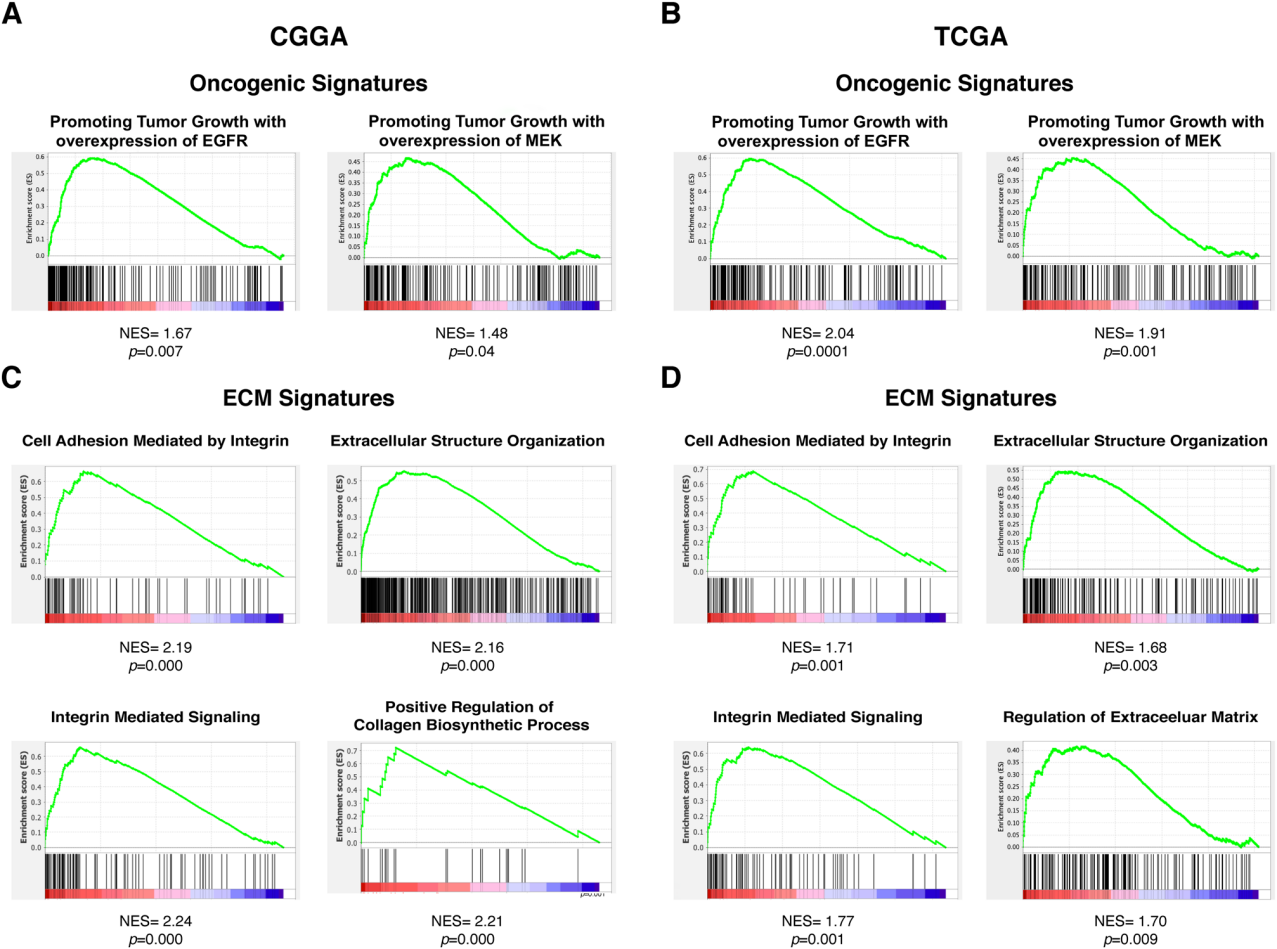
Gene set enrichment analysis (GSEA) for the expression level of PIEZO1 and the signal pathways activated were present. (**A**) GSEA for the expression level of PIEZO1 from CGGA was described relating to oncogenic signatures; (**B**) GSEA for the expression level of PIEZO1 from TCGA was described relating to oncogenic signatures. NES and normalized *p* value were shown at the bottom of each figure.

We next investigated the association of abnormally activated signalling pathways with the expression heterogeneity of PIEZO1 in gliomas. GSEA was used to identify the activated biological pathways. Several oncological signatures were strongly correlated with high PIEZO1 expression in samples from both the CGGA and the GCTA databases (c5.mf.v6.1.symbols). From CGGA, 174/188 gene sets were found to be upregulated in samples expressing PIEZO1 at high levels, among which 129 gene sets were significant at an FDR < 25%, and 82 gene sets were significantly enriched at a nominal *p* value < 5%. From TCGA, 140/187 gene sets were upregulated in high PIEZO1 expression samples, among which 18 gene sets were significant at FDR < 25%, and 18 gene sets were significantly enriched at a nominal *p* value < 5%. Figure 5A,B show the enriched signalling pathways based on their normalized enrichment score (NES). Notably, oncological signatures such as EGFR and MEK showed a strong positive correlation with high PIEZO1 expression. The first 20 oncological signatures that were positively correlated with the expression of PIEZO1 in gliomas from GSEA are shown in Supplemental Table 1.

Next, we used the (c5.bp.v7.0.symbols) gene set from GSEA to identify the activated biological pathways related to ECM regulation. Several signatures were found to strongly correlate with high PIEZO1 expression samples in both CGGA and GCTA. In the CGGA, 3210/3899 gene sets were upregulated in high PIEZO1 expression samples, among which 1999 gene sets were significant at an FDR < 25%, and 1367 gene sets were significantly enriched at a nominal *p* value < 5%. In the TCGA, 2522/3453 gene sets were upregulated in high PIEZO1 expression samples, among which 379 gene sets were significantly enriched at a nominal *p* value < 5%. Figure 5C,D show the enriched signalling pathways based on their normalized enrichment score (NES). Pathways regulating ECM were strongly positively correlated with high PIEZO1 expression samples. The first 20 ECM-regulating signatures that were positively correlated with the expression of PIEZO1 in gliomas from GSEA are shown in Supplemental Table 2.

### PIEZO1 is related to the tumour microenvironment

**Figure 6:**
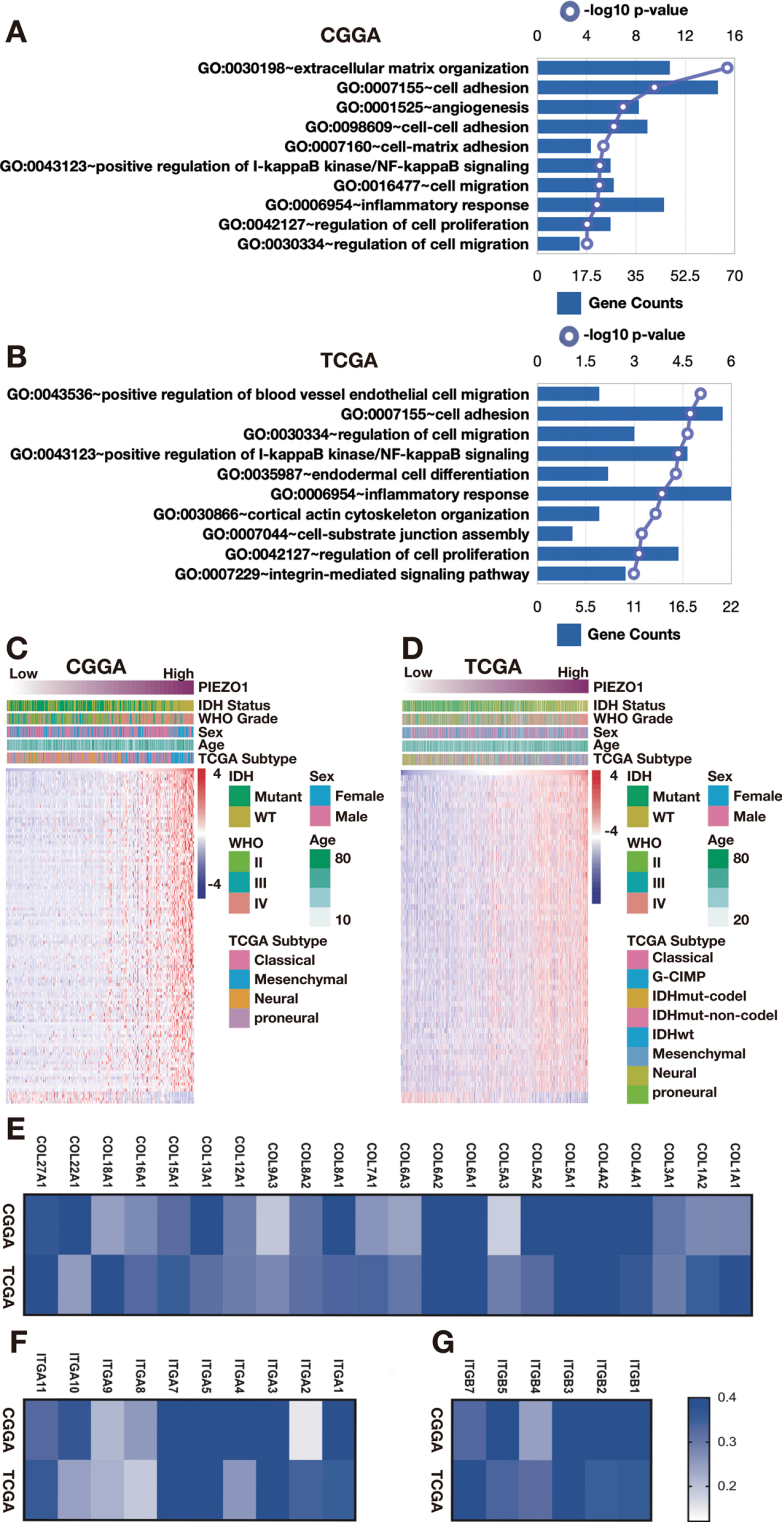
Analysis of genetics of PIEZO1 expression through RNA-seq. (**A**,**B)** Gene ontology analysis of positively related biological process showed that PIEZO1 was mostly in regulating extracellular matrix in CGGA and TCGA datasets; (**C**,**D**) Heatmaps showed that most extracellular matrix-related genes were positively correlated with PIEZO1 expression in CGGA and TCGA datasets, while a small number of genes were negatively associated; (**E**–**G**) Heatmaps indicated that most collagen encoded gene family (COL) and integrin encoded gene family (ITG) were positively correlated with PIEZO1 expression in CGGA and TCGA datasets.

Based on these results, we believe PIEZO1 might play a role in the biological development of gliomas. To explore the correlation between biological function and PIEZO1 expression in gliomas, we selected 392 genes and 368 genes, which were significantly correlated with PIEZO1 using Pearson correlation analysis (Pearson |R|> 0.35) in the TCGA and CGGA datasets, respectively, to conduct an analysis. We used GO analysis in the DAVID Bioinformatics Resources 6.8 to investigate the functions of related genes. As the gene functions were sorted by the *p* value in increasing order in the CGGA dataset, cell microenvironment-related genes that were responsible for ECM organization, cell adhesion, angiogenesis, cell migration and proliferation were positively correlated with PIEZO1 expression (Fig. 6A). Additionally, these results were verified in the TCGA dataset (Fig. 6B).

To investigate the expression of genes associated with PIEZO1 in the microenvironment in gliomas, we included an ECM-related gene set from the GSEA and found 110 genes that were most relevant to PIEZO1 (Pearson |R|> 0.35) in the CGGA database to draw the heatmap. Among the selected genes, 106 genes were positively correlated to PIEZO1 expression, while 4 genes showed negative correlation with PIEZO1 expression (Fig. 6C). In the TCGA database, 61 genes were most relevant to PIEZO1 (Pearson |R|> 0.35). Among the selected genes, 59 genes were positively correlated to PIEZO1 expression, while 2 genes showed a negative correlation with PIEZO1 expression (Fig. 6D). Thus, PIEZO1 is positively correlated with the most relevant ECM genes in gliomas. As described before, the expression of PIEZO1 was high in GBM and highly metastatic mesenchymal subtype gliomas, and it was correlated with cell microenvironment-related genes. These results suggest that PIEZO1 may play an essential role in glioma aggressiveness.

To investigate how PIEZO1 interacts with the tumour microenvironment and regulates glioma malignancy, we performed a computational analysis to identify the specific microenvironment-related genes that were correlated with PIEZO1 expression. Two gene groups, namely, the collagen-encoded gene family (COL) and the integrin-encoded gene family (ITG), are known to regulate ECM remodelling and to generate the cytoskeleton network system, and both are highly expressed along with PIEZO1 (Fig. 6E–G).

### PIEZO1 is relevant to ECM signalling pathways in gliomas

**Figure 7:**
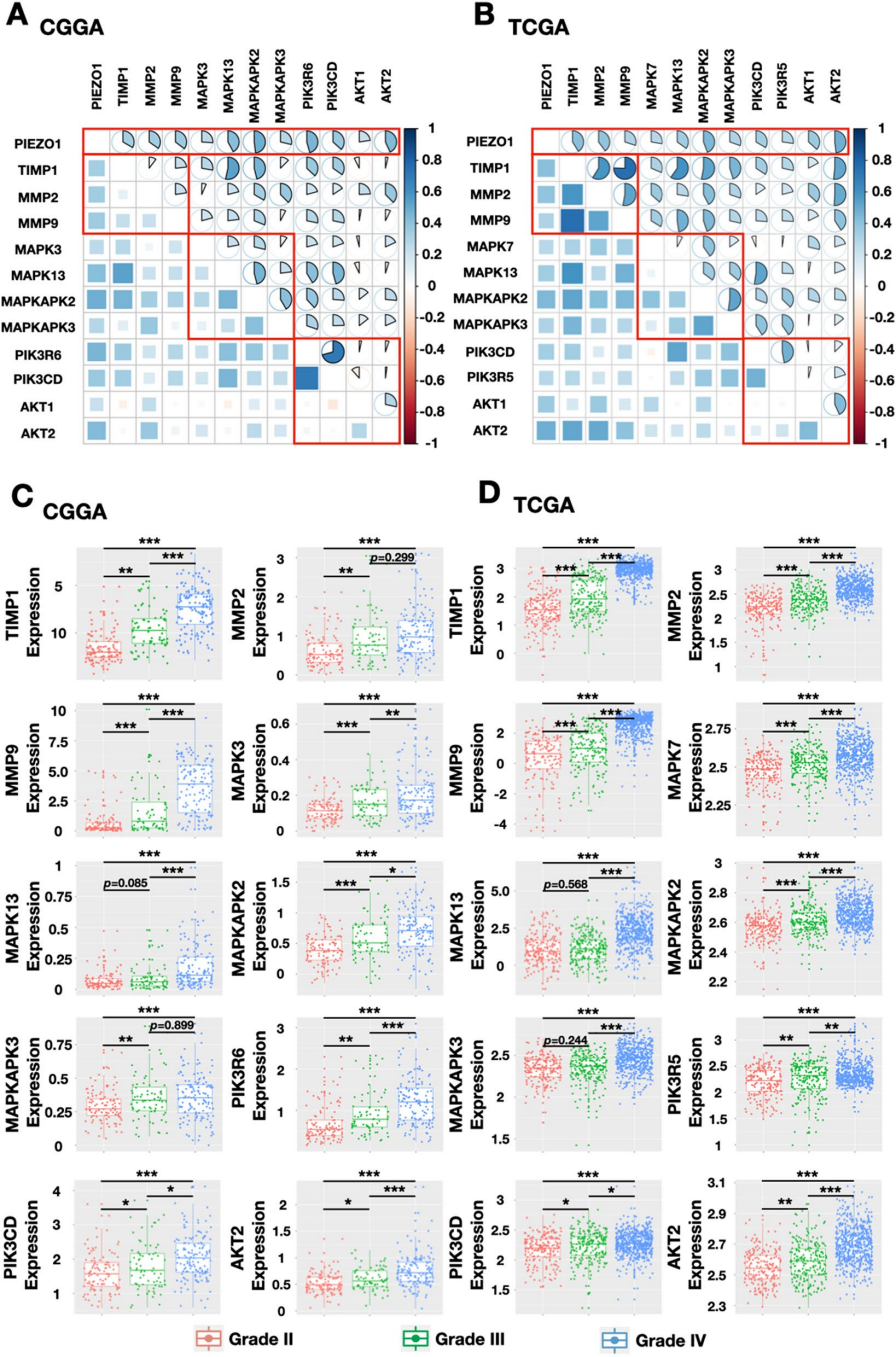
Association of PIEZO1 expression and matrix metalloproteinases (MMP) family, tissue inhibitors of metalloproteinases (TIMP) family, mitogen-activated protein kinase (MAPK) family and Phosphoinositide 3-kinases (PI3Ks) family expression in CGGA and TCGA datasets. (**A**,**B**) Association of PIEZO1 expression and MMP, TIMP, MAPK and PI3K family expression in in CGGA and TCGA datasets; (**C**,**D**) MMP, TIMP, MAPK and PI3K family expression such as TMP1, MMP9, MAPK13, MAPKAPK3 and PIK3CD were upregulated along with the increasing of the WHO grade. ***p < 0.001, **0.001 < *p* < 0.01; * 0.01 < *p* < 0.05.

Among its functions in the tumour microenvironment, PIEZO1 appears to play a particularly strong role in ECM signalling pathways to regulate tumour-associated tissue remodelling. We analysed the matrix metalloproteinase (MMP) family, tissue inhibitors of metalloproteinases (TIMP) family, mitogen-activated protein kinase (MAPK) family and phosphoinositide 3-kinase (PI3Ks) family to clarify the role of PIEZO1 in ECM signal transduction. As shown in Fig. 7A,B, PIEZO1 expression was positively correlated with TIMP1, MMP2, MMP9, MAPK13, MAPKAPK2, PIK3R6, PIK3CD and AKT2 in both the CGGA and TCGA databases. Among these genes, four genes (TIMP1, MAPK13, MAPKAPK2 and MAPKAPK3) are known to regulate cell proliferation, and MMP2 and MMP9 control tumour-associated tissue remodelling; PIK3CD is involved in the immune response, and AKT2 regulates tumourigenesis. Furthermore, we analysed the expression of these ECM-related genes based on the classification of gliomas mentioned before. Strikingly, the expression of all ECM-related genes that were positively correlated with PIEZO1 were significantly higher in glioblastoma (WHO grade IV) compared with low grade gliomas (WHO grade II and WHO grad III, Fig. 7C,D). Taken together, these data indicate that PIEZO1 may provide a physical means for the transfer of mechanical forces, through which the tumour cells are able to directly respond to ECM changes to regulate gene transcription. Meanwhile, the instability of the tumour microenvironment due to ECM changes also allows extra space for tumour proliferation and metastasis.

## Discussion

Gliomas are the most common and lethal primary brain tumours in adults. Limited improvements in outcomes have been made despite extensive study and combination of conventional treatments. New therapeutic targets are urgently needed. Studies on microenvironment-targeted therapeutic strategies have opened a new avenue for tumour treatment^32^. Mechanical cues play a critical role in tumour progression and in shaping the TME by regulating both tumour cells and the TME. PIEZO1 is an intrinsically mechanosensitive ion channel that can interact with many signalling pathways in multiple cancer malignancies^12,13,33^. However, it is unknown whether PIEZO1 regulates glioma development and whether the mechanical environment in tumours interacts with the mechanosensory function of PIEZO1 to promote malignancy in gliomas. Therefore, identification of the molecular and clinical correlations between PIEZO1 expression and glioma malignancy will facilitate the establishment of a potential therapeutic target and shed light on glioma treatment.

In this study, we analysed PIEZO1 expression in glioma. High levels of PIEZO1 expression showed an association with malignant entities, including a high WHO Grade, IDH wild-type status and mesenchymal subtype gliomas. The most important common characteristic among gliomas with a high WHO grade, IDH wild-type status and mesenchymal subtype is aggressiveness. It has been reported that tissue stiffness in human low to high grade gliomas gradually increased from 100 to 10^4^ Pa^34^ and that tumour stiffness correlates with aggressiveness^35^. These results suggest that increasing intra-tumoural solid pressure developed by high grade glioma provides mechanical cues that stimulate PIEZO1, which further plays an essential role in tumour cell proliferation and metastasis. Moreover, we also found that a high level of PIEZO1 was predictive of a poor prognosis. This is the first study to present the PIEZO1 expression pattern in gliomas based on different grading systems including 2016 WHO molecular classifications and the WHO Grade system according to the CGGA dataset and be verified by the TCGA dataset.

To investigate molecular mechanism of glioma, we analysed the distinct genomic alternations in order of increasing PIEZO1 expression. The data show a positive correlation between somatic mutations and PIEZO1 expression. Tumour suppressor genes, including PTEN and NF1, were detected at high amplification peaks with high PIEZO1 expression. The oncogenic driver EGFR was found to be enriched with high expression of PIEZO1. It has been reported that tumour cell microenvironment stiffness regulates tumour development by interacting with EGFR-dependent signalling in glioma^36,37^. In addition, genomic alternations may also contribute to regulating the TME and promoting tumour cell proliferation^38^. Therefore, PIEZO1 appears to take part in the glioma aggressive pathophysiological process, and it can act as the essential biomarker for glioma malignancy and provides a potential therapeutic target to overcome glioma.

As for the biological function of PIEZO1 expression in gliomas, we found that cell microenvironment-related genes that are related to ECM organization, cell adhesion, angiogenesis, cell migration and proliferation were positively correlated with PIEZO1 expression. Additionally, two gene groups, the collagen-encoded gene family (COL) and the integrin-encoded gene family (ITG) that are known to regulate ECM remodelling and compose the cytoskeleton network system were positively correlated with PIEZO1 expression^39,40^. These results indicate that PIEZO1 regulates glioma aggressiveness through its biological function in ECM remodelling. ECM components are major molecules in the cell microenvironment^36^. In solid tumours, the ECM has the capacity to drive tumour aggressiveness^3^. It has been reported that PIEZO1 can activate integrins intracellularly^41^. As the key ECM adhesion receptors, integrins interact with the integrin-FAK signalling pathway to regulate cancer cell behaviour, promoting migration, proliferation and survival^36^. Integrin inhibitors (e.g., cilengitide or etaracizumab) have been tested in clinical trials^42–45^. A randomized phase II trial showed that cilengitide monotherapy was well tolerated and noted a modest anti-tumour activity in recurrent glioblastoma^45^. However, a phase III trial showed that combination of cilengitide with standard temozolomide chemoradiotherapy had no benefit over conventional therapy in glioblastoma, particularly in tumours with methylated MGMT promoter^42^. Cilengitide as a subunit antagonist of the integrins αvβ3 and αvβ5 remains controversial^42^. PIEZO1 is the upstream molecule of integrins and can directly sense the mechanical changes caused by tumour tissue pressure, which makes PIEZO1 a potential biomarker as a therapeutic target.

The molecular mechanisms through which PIEZO1 regulates glioma malignancy involve multiple signalling pathways. As shown in the results, PIEZO1 expression is positively correlated with genes that are responsible for cell proliferation, tumour-associated tissue remodelling, immune response and tumourigenesis. These data indicate that PIEZO1 acts as a central functional point to form an integrated mechanical-regulation molecule network, here giving rise to the hypothesis that a feedback mechanism where the increasing tumour tissue stiffness caused by abnormal regulation of cell growth activates the PIEZO1 ion channel and multiple ECM-related pathways that are downstream, in turn modulating cell proliferation and TME remodelling to increase tissue pressure. This also explains why in high grade gliomas such as GBM, PIEZO1 expression is expressed at a high level. Meanwhile, cytoskeletal dynamics are also associated with PIEZO1 expression. A loose supportive structure due to loss of collagen in the TME provides more physical space for tumour cells. In parallel, tumours with a remodelled TME are able to compress blood supply through exerting physical forces on their microenvironment. Hypoperfusion and hypoxia lower the efficacy of tumour targeted drug delivery, promoting immune evasion and tumour metastasis. Evidence has shown that expression of PIEZO1 increased in multiple malignant diseases including bladder carcinoma, synovial sarcoma, osteosarcoma, breast cancer and gastric cancer^12,46–49^. PIEZO1 is also involved in 2D cell migration and 3D invasion in the MCF-7 cell line, a gastric cancer cell line, and small-cell lung carcinoma cell lines^12,33,49^. Taken together, PIEZO1 not only regulates tumour cell proliferation but modulates the TME as well to promote tumour cell metastasis.

As expression of PIEZO1 is positively associated with gliomas, pharmacological inhibition of PIEZO1 might prove an efficacious strategy in treating these malignant gliomas. Although current identified inhibitors (ruthenium red, Gd3^+^ and Grammostola spatulata spider toxin GsMTx4) are not PIEZO1-specific, it has been reported that the use of GsMTx4 may inhibit PIEZO1 activity in the urothelium, affect PIEZO1-dependent arterial remodelling and reduce cell death due to high strain mechanical injury^50–52^. Because PIEZO1 plays a vital role in an abundance of physiological processes, more research needs to be done to investigate tumour-specific PIEZO1 knockdown and provide a potential tumour cell and TME-targeted therapy.

Our findings indicate that PIEZO1 might play a role as a biomarker in gliomas. The biological significance of PIEZO1 was explored by analysing two large sample-sized glioma cohorts. PIEZO1 expression is correlated with increasing tumour tissue stiffness as tumourigenesis proceeds, and the function of PIEZO1 aggravates tumour cell proliferation and glioma malignancy. Furthermore, the tumour-specific inhibitor of PIEZO1 needs to be addressed in future studies. However, we still need to clarify the specific molecular mechanism of PIEZO1-related mechanical–biological transduction signalling pathways and how to modulate these molecules to regulate glioma progression.

In conclusion, using publicly available transcriptomic and genomic profiling data, we found that PIEZO1 was upregulated in high grade glioma and predictive of a poor prognosis. Additionally, PIEZO1 was positively correlated with microenvironment remodelling-related genes and appears to function as a central checkpoint of ECM remodelling pathways. These findings establish PIEZO1 as a novel focus to study the mechanism of glioma malignancy and as a potential therapeutic target.

## Supplementary information

Supplementary Figure 1.

Supplementary Figure 2.

Supplementary Legends.

Supplementary Table 1.

Supplementary Table 2.
