# Supplementary figures and images for: Identification of PIEZO1 as a potential prognostic marker in gliomas
Source: Sci Rep. 2020 Sep 30;10:16121. doi: 10.1038/s41598-020-72886-8 (PMC7528027; doi:10.1038/s41598-020-72886-8)

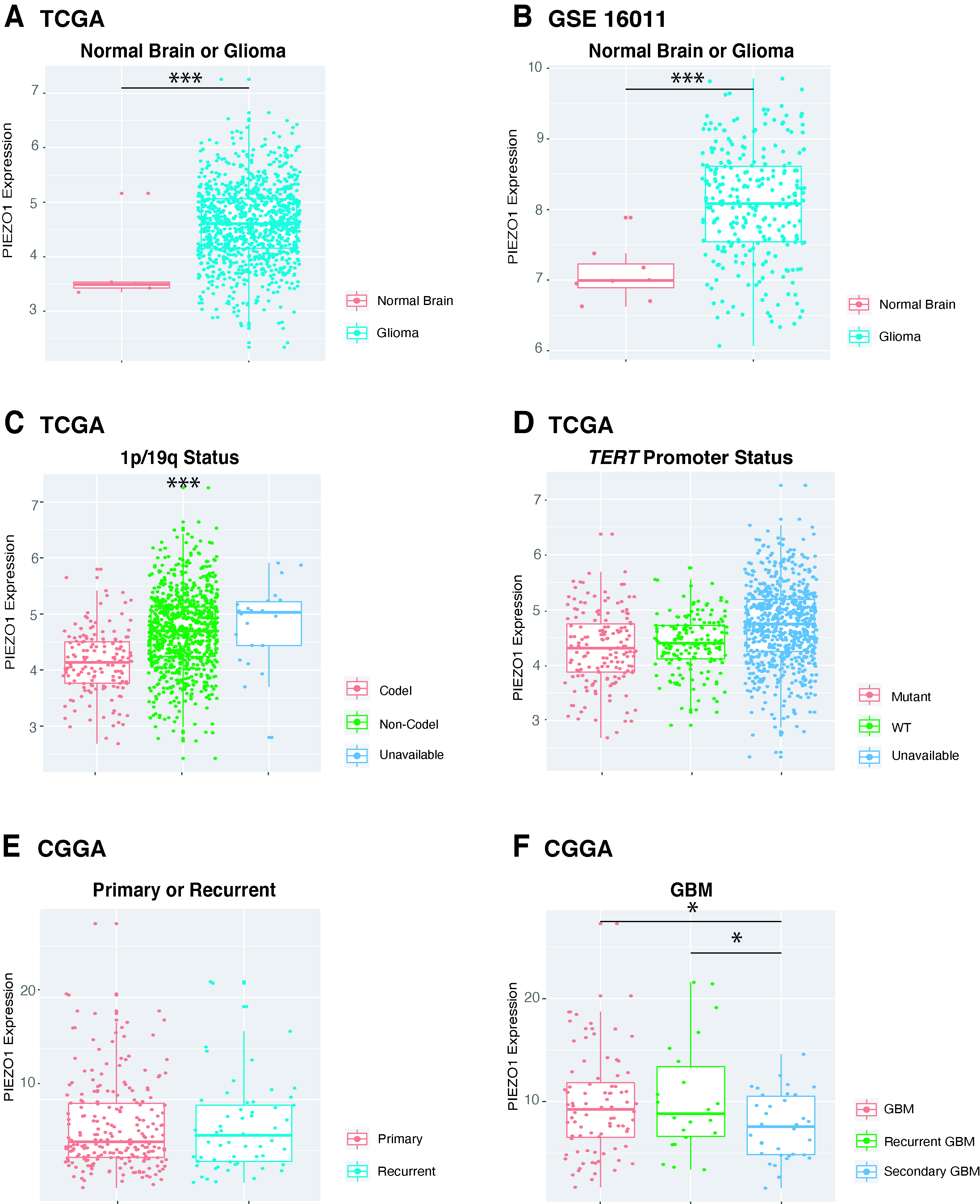

Supplement: Supplementary file 1 — Supplementary Figure 1. [file 41598_2020_72886_MOESM1_ESM.tif]

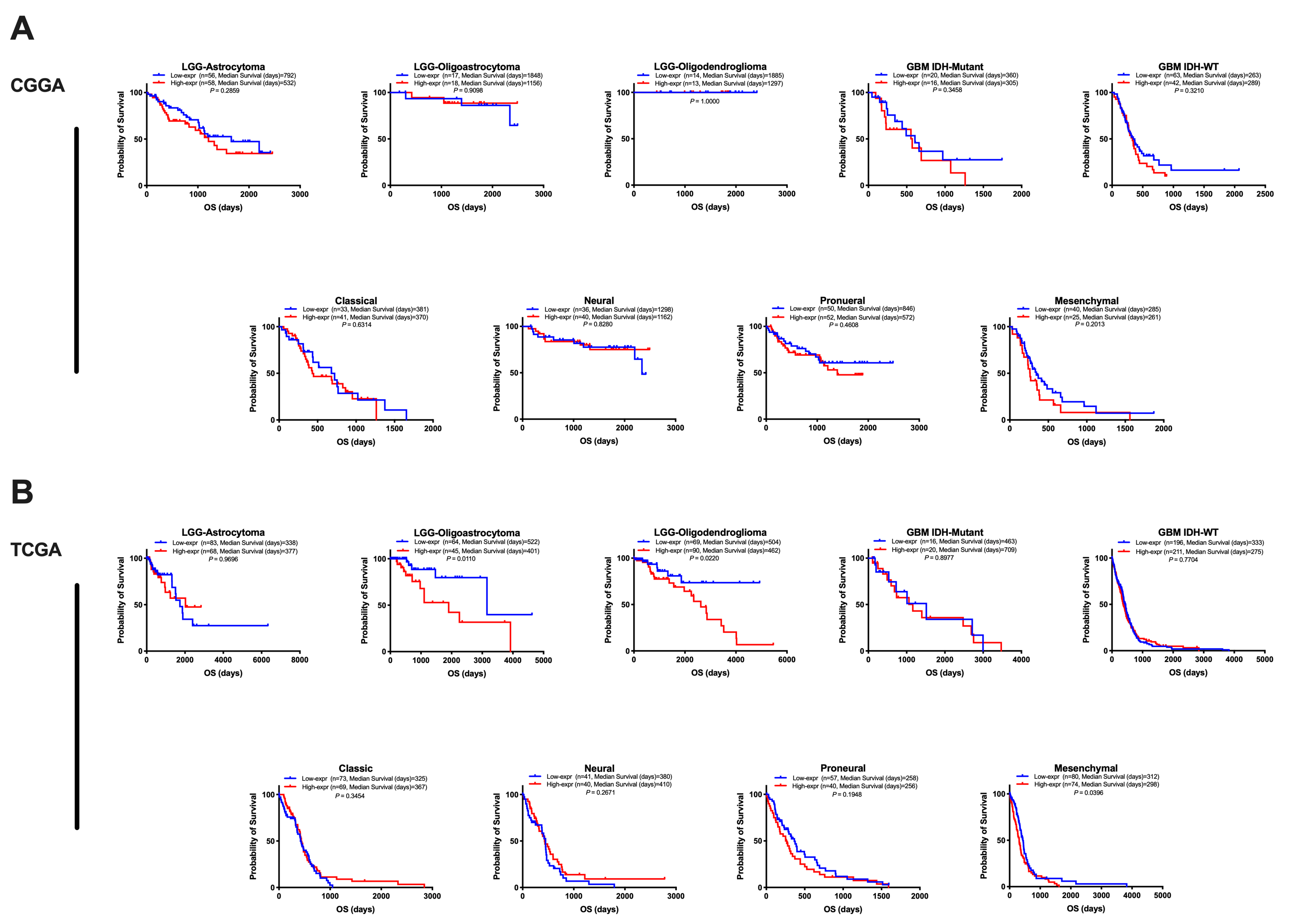

Supplement: Supplementary file 2 — Supplementary Figure 2. [file 41598_2020_72886_MOESM2_ESM.tiff]
