# Supplementary material for: Identification of PIEZO1 as a potential prognostic marker in gliomas
Source: Sci Rep. 2020 Sep 30;10:16121. doi: 10.1038/s41598-020-72886-8 (PMC7528027; doi:10.1038/s41598-020-72886-8)
Supplement: Supplementary file 3 — Supplementary Legends. [file 41598_2020_72886_MOESM3_ESM.docx]

**Identification of PIEZO1 as a Potential Prognostic Marker in Gliomas**

## Wenjianlong Zhou*, MD^1,2^, Xiangxiang Liu*, MD^3,4^, Jan Willem Maurits van Wijnbergen, MD, PhD^2^, Linhao Yuan, MD^1^, Yuan Liu, PhD^4^，Chuanbao Zhang, MD^1^, Wang Jia, MD^1,5,6^

1Department of Neurosurgery, Beijing Tiantan Hospital, Capital Medical University, Beijing, China;

2Edwin L. Steele Laboratories, Department of Radiation Oncology, Massachusetts General Hospital, Harvard Medical School, Boston, MA, United States of America;

3Beijing Tongren Eye Center, Beijing Tongren Hospital, Capital Medical University, Beijing, China;

4Bascom Palmer Eye Institute, University of Miami Miller School of Medicine, Miami, FL, United States of America;

5China National Clinical Research Center for Neurological Diseases (NCRC-ND), Beijing, China;

6Beijing Neurosurgical Institute, Beijing, China

***These authors contributed equally to this work.**

**Corresponding author**

**Wang Jia:** [**jwttyy@126.com**](mailto:jwttyy@126.com) **and Chuanbao Zhang:chuanbao123@126.com**

**Supplemental Figure Legends**

**Supplementary Fig S1. The additional landscape of clinical and molecular features in associations with PIEZO1 expression.** A:PIEZO1 expression in normal brain tissues and gliomas in TCGA; B:PIEZO1 expression in normal brain tissues and gliomas in GSE 16011; C: The expression levels of PIEZO1 increased in Non-Codel group according to 1p/19q status in TCGA; D: The expression levels of PIEZO1 according to TERT promoter status in TCGA; E: The expression levels of PIEZO1 according to primary or recurrent status in CGGA; F: PIEZO1 expression increased in GBM and recurrent GBM than secondary GBM. *** indicates *p* < 0.001, ** indicates 0.001 < *p* < 0.01, * indicates 0.01 < *p* < 0.05.

**Supplementary Fig S2.** **The prognostic value of PIEZO1 expression in stratified analysis.** Kaplan – Meier survival analysis was performed. Blue lines show the survival curve in patients of low PIEZO1 expression; Red lines show the survival curve in patients of high PIEZO1 expression.

**Supplementary Table S1. Oncogenic Signatures Enrichment in High Expression of PIEZO 1 Phenotype**

**Supplementary Table S2. ECM Signatures Enrichment in High Expression of PIEZO 1 Phenotype**
