# Supplementary material for: Identification of PIEZO1 as a potential prognostic marker in gliomas
Source: Sci Rep. 2020 Sep 30;10:16121. doi: 10.1038/s41598-020-72886-8 (PMC7528027; doi:10.1038/s41598-020-72886-8)
Supplement: Supplementary file 4 — Supplementary Table 1. [file 41598_2020_72886_MOESM4_ESM.docx]

| **S1 Table. Oncogenic Signatures Enrichment in High Expression of PIEZO 1 Phenotype** | | | | |
| --- | --- | --- | --- | --- |
| **Database** | **Pathway** | **Size** | **NES** | **Normalized *p* value** |
| **CGGA** | | | | |
|  | MEL18_DN.V1_UP | 136 | 2.158322 | <0.0001 |
|  | BMI1_DN.V1_UP | 141 | 2.0719633 | <0.0001 |
|  | ESC_V6.5_UP_EARLY.V1_DN | 169 | 2.0576894 | <0.0001 |
|  | GCNP_SHH_UP_LATE.V1_DN | 172 | 2.0556903 | <0.0001 |
|  | EGFR_UP.V1_UP | 189 | 2.0433402 | <0.0001 |
|  | IL15_UP.V1_UP | 174 | 2.0176651 | <0.0001 |
|  | SNF5_DN.V1_UP | 165 | 1.9952221 | <0.0001 |
|  | BMI1_DN_MEL18_DN.V1_UP | 137 | 1.9742163 | <0.0001 |
|  | BCAT_BILD_ET_AL_UP | 45 | 1.9684246 | 0.0019 |
|  | E2F1_UP.V1_DN | 185 | 1.9477105 | <0.0001 |
|  | IL2_UP.V1_UP | 180 | 1.9399532 | <0.0001 |
|  | MEK_UP.V1_UP | 187 | 1.9102908 | 0.0020 |
|  | CAHOY_ASTROGLIAL | 93 | 1.907971 | 0.0041 |
|  | LEF1_UP.V1_UP | 192 | 1.9077953 | 0.0059 |
|  | ESC_J1_UP_EARLY.V1_DN | 172 | 1.8286176 | <0.0001 |
|  | STK33_SKM_UP | 266 | 1.8242611 | 0.0020 |
|  | KRAS.DF.V1_UP | 185 | 1.812265 | 0.0020 |
|  | RAF_UP.V1_UP | 187 | 1.810586 | 0.0020 |
|  | AKT_UP.V1_DN | 183 | 1.8099985 | 0.0020 |
|  | RB_DN.V1_DN | 123 | 1.7982708 | 0.0040 |
| **TCGA** | | | | |
|  | MEL18_DN.V1_UP | 123 | 1.7590766 | <0.0001 |
|  | BMI1_DN.V1_UP | 127 | 1.7457957 | 0.0040 |
|  | EGFR_UP.V1_UP | 172 | 1.6793314 | 0.0080 |
|  | ESC_V6.5_UP_EARLY.V1_DN | 138 | 1.6500908 | 0.0120 |
|  | PRC2_EED_UP.V1_DN | 164 | 1.6077037 | 0.0165 |
|  | BCAT_BILD_ET_AL_UP | 35 | 1.600844 | 0.0175 |
|  | IL2_UP.V1_UP | 142 | 1.5916027 | 0.0116 |
|  | BMI1_DN_MEL18_DN.V1_UP | 121 | 1.5855479 | 0.0163 |
|  | CORDENONSI_YAP_CONSERVED_SIGNATURE | 53 | 1.5666053 | 0.0122 |
|  | LEF1_UP.V1_UP | 165 | 1.5547578 | 0.0102 |
|  | IL15_UP.V1_UP | 140 | 1.541341 | 0.0156 |
|  | ESC_J1_UP_EARLY.V1_DN | 138 | 1.5197234 | 0.0196 |
|  | CYCLIN_D1_KE_.V1_UP | 149 | 1.5114262 | 0.0202 |
|  | CSR_EARLY_UP.V1_UP | 143 | 1.5002155 | 0.0591 |
|  | ESC_J1_UP_LATE.V1_UP | 147 | 1.49522 | 0.0337 |
|  | MEK_UP.V1_UP | 168 | 1.484115 | 0.0437 |
|  | ESC_V6.5_UP_LATE.V1_DN | 141 | 1.4709296 | 0.0165 |
|  | RB_P107_DN.V1_UP | 116 | 1.442355 | 0.1066 |
|  | RB_DN.V1_DN | 108 | 1.4418675 | 0.0659 |
|  | CAHOY_ASTROGLIAL | 90 | 1.4407688 | 0.0703 |
