# Supplementary material for: Identification of PIEZO1 as a potential prognostic marker in gliomas
Source: Sci Rep. 2020 Sep 30;10:16121. doi: 10.1038/s41598-020-72886-8 (PMC7528027; doi:10.1038/s41598-020-72886-8)
Supplement: Supplementary file 5 — Supplementary Table 2. [file 41598_2020_72886_MOESM5_ESM.docx]

| **S2 Table. ECM Signatures Enrichment in High Expression of PIEZO 1 Phenotype** | | | | |
| --- | --- | --- | --- | --- |
| **Database** | **Pathway** | **Size** | **NES** | **Normalized *p* value** |
| **CGGA** | | | | |
|  | GO_INTEGRIN_MEDIATED_SIGNALING_PATHWAY | 91 | 2.24 | <0.0001 |
|  | GO_POSITIVE_REGULATION_OF_COLLAGEN_BIOSYNTHETIC_PROCESS | 23 | 2.21 | <0.0001 |
|  | GO_SUBSTRATE_ADHESION_DEPENDENT_CELL_SPREADING | 81 | 2.20 | <0.0001 |
|  | GO_ENDOTHELIAL_CELL_MIGRATION | 199 | 2.19 | <0.0001 |
|  | GO_CELL_ADHESION_MEDIATED_BY_INTEGRIN | 64 | 2.19 | <0.0001 |
|  | GO_FORMATION_OF_PRIMARY_GERM_LAYER | 111 | 2.18 | <0.0001 |
|  | GO_AMINOGLYCAN_BIOSYNTHETIC_PROCESS | 106 | 2.17 | <0.0001 |
|  | GO_BLOOD_VESSEL_ENDOTHELIAL_CELL_MIGRATION | 119 | 2.16 | <0.0001 |
|  | GO_AMINOGLYCAN_METABOLIC_PROCESS | 126 | 2.14 | <0.0001 |
|  | GO_LYMPHOCYTE_APOPTOTIC_PROCESS | 68 | 2.13 | <0.0001 |
|  | GO_REGULATION_OF_ENDOTHELIAL_CELL_MIGRATION | 155 | 2.13 | <0.0001 |
|  | GO_EXTRACELLULAR_STRUCTURE_ORGANIZATION | 398 | 2.12 | <0.0001 |
|  | GO_POSITIVE_REGULATION_OF_VASCULATURE_DEVELOPMENT | 177 | 2.11 | <0.0001 |
|  | GO_POSITIVE_REGULATION_OF_ENDOTHELIAL_CELL_MIGRATION | 95 | 2.10 | <0.0001 |
|  | GO_NEGATIVE_REGULATION_OF_MULTI_ORGANISM_PROCESS | 168 | 2.10 | <0.0001 |
|  | GO_MEMBRANE_PROTEIN_ECTODOMAIN_PROTEOLYSIS | 41 | 2.10 | <0.0001 |
|  | GO_ENDODERM_DEVELOPMENT | 72 | 2.09 | <0.0001 |
|  | GO_POSITIVE_REGULATION_OF_NF_KAPPAB_TRANSCRIPTION_FACTOR_ACTIVITY | 146 | 2.09 | <0.0001 |
|  | GO_CELL_JUNCTION_ASSEMBLY | 231 | 2.09 | <0.0001 |
|  | GO_CELL_JUNCTION_ORGANIZATION | 270 | 2.09 | <0.0001 |
| **TCGA** | | | | |
|  | GO_POSITIVE_REGULATION_OF_CELL_JUNCTION_ASSEMBLY | 28 | 1.91 | <0.0001 |
|  | GO_POSITIVE_REGULATION_OF_FOCAL_ADHESION_ASSEMBLY | 21 | 1.90 | <0.0001 |
|  | GO_POSITIVE_REGULATION_OF_ADHERENS_JUNCTION_ORGANIZATION | 27 | 1.84 | 0.0038 |
|  | GO_GROWTH_PLATE_CARTILAGE_CHONDROCYTE_DIFFERENTIATION | 20 | 1.83 | <0.0001 |
|  | GO_POSITIVE_REGULATION_OF_PROTEIN_LOCALIZATION_TO_NUCLEUS | 60 | 1.83 | <0.0001 |
|  | GO_RENAL_SYSTEM_VASCULATURE_DEVELOPMENT | 22 | 1.78 | 0.0059 |
|  | GO_INTEGRIN_MEDIATED_SIGNALING_PATHWAY | 78 | 1.77 | 0.0020 |
|  | GO_SUBSTRATE_ADHESION_DEPENDENT_CELL_SPREADING | 63 | 1.77 | <0.0001 |
|  | GO_CHONDROCYTE_DIFFERENTIATION_INVOLVED_IN_ENDOCHONDRAL_BONE_MORPHOGENESIS | 25 | 1.75 | 0.0039 |
|  | GO_VASCULAR_ENDOTHELIAL_GROWTH_FACTOR_RECEPTOR_SIGNALING_PATHWAY | 82 | 1.74 | 0.0140 |
|  | GO_GROWTH_PLATE_CARTILAGE_MORPHOGENESIS | 16 | 1.74 | 0.0019 |
|  | GO_CHONDROCYTE_DEVELOPMENT_INVOLVED_IN_ENDOCHONDRAL_BONE_MORPHOGENESIS | 20 | 1.72 | 0.0039 |
|  | GO_ERYTHROCYTE_HOMEOSTASIS | 90 | 1.72 | 0.0042 |
|  | GO_REGULATION_OF_SUBSTRATE_ADHESION_DEPENDENT_CELL_SPREADING | 35 | 1.72 | 0.0021 |
|  | GO_PLACENTA_BLOOD_VESSEL_DEVELOPMENT | 29 | 1.72 | 0.0098 |
|  | GO_GROWTH_PLATE_CARTILAGE_DEVELOPMENT | 28 | 1.72 | 0.0019 |
|  | GO_ENDODERM_FORMATION | 42 | 1.72 | 0.0060 |
|  | GO_CELL_ADHESION_MEDIATED_BY_INTEGRIN | 54 | 1.71 | 0.0020 |
|  | GO_ENDOCHONDRAL_BONE_MORPHOGENESIS | 59 | 1.71 | 0.0117 |
|  | GO_MYELOID_CELL_HOMEOSTASIS | 112 | 1.71 | 0.0041 |
